# Safety of neuromuscular electrical stimulation among critically ill patients: systematic review

**DOI:** 10.5935/0103-507X.20180036

**Published:** 2018

**Authors:** Amanda Sachetti, Marta Fiorvanti Carpes, Alexandre Simões Dias, Graciele Sbruzzi

**Affiliations:** 1 Curso de Medicina, Escola de Saúde, IMED - Passo Fundo (RS), Brasil.; 2 Universidade Federal do Rio Grande do Sul - Porto Alegre (RS), Brasil.; 3 Hospital de Clínicas de Porto Alegre, Universidade Federal do Rio Grande do Sul - Porto Alegre (RS), Brasil.

**Keywords:** Safety, Electric stimulation therapy, Respiration, artificial, Drug-related side effects and adverse reactions, Physical stimulation, Intensive care units

## Abstract

**Objective:**

To review the evidence on the safety of neuromuscular electrical stimulation when used in the intensive care unit.

**Methods:**

A systematic review was conducted; a literature search was performed of the MEDLINE (via PubMed), PEDro, Cochrane CENTRAL and EMBASE databases, and a further manual search was performed among the references cited in randomized studies. Randomized clinical trials that compared neuromuscular electrical stimulation to a control or placebo group in the intensive care unit and reporting on the technique safety in the outcomes were included. Hemodynamic variables and information on adverse effects were considered safety parameters. Articles were independently analyzed by two reviewers, and the data analysis was descriptive.

**Results:**

The initial search located 1,533 articles, from which only four randomized clinical trials were included. Two studies assessed safety based on hemodynamic variables, and only one study reported an increase in heart rate, respiratory rate and blood lactate, without clinical relevance. The other two studies assessed safety based on reported adverse effects. In one, 15% of patients described a prickling sensation, without any clinically relevant abnormalities. In the other, one patient suffered a superficial burn due to improper parameter configuration.

**Conclusion:**

Neuromuscular electrical stimulation is safe for critically ill patients; however, it should be applied by duly trained professionals and with proper evidence-based parameters.

## INTRODUCTION

The survival rate of critically ill patients has increased over time as a function of technological advances and new techniques used for providing intensive care.^(^^1^^)^ However, in parallel with such an increase in the survival rate, the therapeutic resources that contribute to such outcomes also cause some comorbidities, such as muscle weakness derived from the loss of muscle mass and strength.^(^^2^^)^ In addition to these factors, one might also mention immobility in the bed, which increases muscle catabolism and reduces the synthesis of proteins and muscle mass.^(^^3^^)^ These muscle disorders might have a negative impact on the patients' independence and quality of life as well as on their functional capacity after discharge from the hospital.^(^^4^^)^

For patients unable to perform active movements, neuromuscular electrical stimulation (NMES) might represent a therapeutic option to increase or maintain their muscle strength. NMES programs seem to be acceptable to patients and result in the improvement of muscle function, exercise capacity and quality of life.^(^^5^^)^ However, estimates of NMES efficacy based on individual studies lack power and precision.^(^^4^^)^

According to some studies, NMES was shown to be effective in the acute stage of a disease,^(^^6^^,^^7^^)^ while in others, it was shown to have no effect in reverting the loss of muscle strength in the acute stage.^(^^8^^,^^9^^)^ Recent studies with variable methodological designs have shown that NMES is safe, feasible and beneficial for patients admitted to the intensive care unit (ICU).^(^^10^^-^^13^^)^ However, the available data are still inconclusive due to the heterogeneity of protocols and the small sample sizes.

The aim of the present systematic review was to investigate the safety of NMES among critically ill patients by comparison to control or placebo groups.

## METHODS

The present systematic review followed the *Preferred Reporting Items for Systematic Reviews and Meta-Analyses* (PRISMA) statement^(^^14^^)^ and was registered in the *International prospective register of systematic reviews* (PROSPERO) on July 18, 2016, under registration number 42016043079.

### Eligibility criteria

Randomized clinical trials (RCT) involving patients admitted to the ICU, under invasive mechanical ventilation and subjected to NMES on the peripheral muscles were included. These patients were compared to a control group, composed of patients receiving other types of physical therapy, no intervention or sham NMES.

The outcome assessed was the safety of NMES among critically ill patients, based on the presence/absence of adverse effects and/or hemodynamic parameters.

The exclusion criteria were pilot RCTs and studies with missing data or without control group data.

### Search strategy

The search was conducted in the following electronic databases: MEDLINE (via PubMed), Physiotherapy Evidence Database (PEDro), Cochrane Central Register of Controlled Trials (CENTRAL) and EMBASE. In addition, a manual search of the references cited in published studies was also performed. The search was performed in October 2016 with the following keywords and corresponding synonyms: "*critical illness*", "*intensive care*", "*intensive care units*", "*electric stimulation*" and "*electric stimulation therapy*". These terms were associated with a sensitive list of terms to locate RCTs.^(^^15^^)^ The full search strategy used for the PubMed database is described in table 1. The search had no language or date limits.

**Table 1:** Search strategy used for PubMed

#1	("Critical Illness"[Mesh] OR “Critical Illness” OR “Critical Illnesses” OR “Illness, Critical” OR “Illnesses, Critical” OR “Critically Ill” OR "Intensive Care"[Mesh] OR "Intensive Care" OR “Care, Intensive” OR “Surgical Intensive Care” OR “Care, Surgical Intensive” OR “Intensive Care, Surgical” OR "Intensive Care Units"[Mesh] OR "Intensive Care Units" OR “Care Unit, Intensive” OR “Care Units, Intensive” OR “Intensive Care Unit” OR “Unit, Intensive Care” OR “Units, Intensive Care” OR “critical illness polyneuromyopathy” OR “Polyneuropathy, Critical Illness” OR “Critical Illness Polyneuropathies” OR “Critical Illness Polyneuropathy” OR “Polyneuropathies, Critical Illness”)
	
#2	("Electric Stimulation"[Mesh] OR “Electrical Stimulation” OR “Electrical Stimulations” OR “Stimulation, Electrical” OR “Stimulations, Electrical” OR “Stimulation, Electric” OR “Electric Stimulations” OR “Stimulations, Electric” OR “Electric Stimulation Therapy"[Mesh] OR “Electric Stimulation Therapy" OR “Therapeutic Electric Stimulation” OR “Electric Stimulation, Therapeutic” OR “Stimulation, Therapeutic Electric” OR “Therapy, Electric Stimulation” OR “Stimulation Therapy, Electric” OR “Electrotherapy” OR “Neuromuscular electrical stimulation” OR “neuromuscular electric stimulation” OR “Electrical muscle stimulation”)
	
#3	((randomized controlled trial[pt] OR controlled clinical trial[pt] OR randomized controlled trials[mh] OR random allocation[mh] OR double-blind method[mh] OR single-blind method[mh] OR clinical trial[pt] OR clinical trials[mh] OR ("clinical trial"[tw]) OR ((singl*[tw] OR doubl*[tw] OR trebl*[tw] OR tripl*[tw]) AND (mask*[tw] OR blind*[tw])) OR ("latin square"[tw]) OR placebos[mh] OR placebo*[tw] OR random*[tw] OR research design[mh:noexp] OR follow-up studies[mh] OR prospective studies[mh] OR cross-over studies[mh] OR control*[tw] OR prospectiv*[tw] OR volunteer*[tw]) NOT (animal[mh] NOT human[mh]))
	
#4	((#1) AND #2) AND #3

### Study selection and data extraction

The titles and abstracts of all the retrieved articles were independently analyzed by two reviewers. Articles whose abstracts did not provide sufficient information were selected for full-text analysis. Following selection based on titles and abstracts, the same reviewers independently selected articles based on full-text analysis; instances of disagreement were solved by consensus.

Data extraction was performed in duplicate by the same two reviewers, who used a standardized form for this purpose. The main outcome was the presence of adverse effects; a second outcome of interest was changes in hemodynamic variables.

### Assessment of risk of bias

The methodological quality of the studies was descriptively assessed by two reviewers according to the method formulated by the Cochrane Collaboration.^(^^16^^)^ The following aspects were considered: selection bias (random sequence generation and allocation concealment), performance bias (blinding of participants and professionals), detection bias (blinding of outcome assessors), attrition bias (incomplete outcome data), reporting bias (selective reporting) and other sources of bias.

### Data analysis

The data were subjected to descriptive and qualitative analysis and are presented in figures and tables.

## RESULTS

### Description of studies

**Figure 1:**
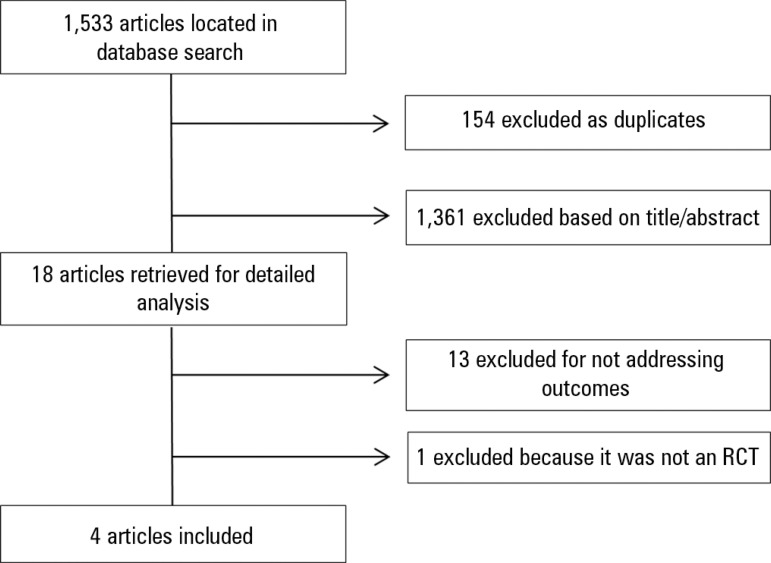
Flowchart representing article search and selection. RCT - randomized clinical trial.

The initial search located 1,533 articles, out of which 18 were rated as potentially relevant and analyzed in detail. Following full-text analysis, 13 articles were excluded for not addressing the outcomes of interest,^(^^5^^-^^7^^,^^9^^,^^17^^-^^24^^)^ and one because it was not an RCT.^(^^1^^)^ The reviewers independently rated four articles as adequate, which together included 162 patients (Table 2, Figure 1).

**Table 2:** Description of selected studies

Study	Groups	Patients (n)	Main objective of study	Intervention parameters	Outcome for safety
Rodriguez et al.^(^^23^^)^	G1: NMES on one side of the body (contralateral side as control)	Total: 16	To assess the effects of NMES on the muscle strength of patients with sepsis under IMV	Frequency 100Hz; pulse duration 300µs; amplitude 20 - 200v; biphasic impulse; intensity controlled by visible or palpable contraction; stimulus applied twice per day for 30 minutes Brachial biceps and quadriceps vastus medialis muscles Application from IMV day 2 until extubation	Adverse effects: superficial burn in a single patient after the first NMES session due to improper configuration
Abu-Khaber et al.^(^^25^^)^	G1: conventional treatment G2: conventional treatment + NMES	Total: 80 G1: 40 G2: 40	To assess the effects of NMES on the peripheral muscles of critically ill patients	Frequency 50Hz; pulse duration 200µs; biphasic symmetrical impulse; duration 15 seconds (1 second rise and 1 second fall); intensity controlled by visible or palpable contraction; stimulus applied once per day for 60 minutes Bilateral quadriceps Application from IMV day 2 to ICU discharge	Adverse effects: six patients (15%) reported a prickling sensation, which was not clinically significant
Akar et al.^(^^26^^)^	G1: active mobilization + NMES G2: NMES G3: active mobilization	Total: 30 G1: 10 G2: 10 G3: 10	To compare the efficacy of active mobilization, active mobilization + NMES and NMES alone on muscles, ventilation weaning and NMES response to inflammation among critically ill patients with COPD	Frequency 50Hz; amplitude 20mA and 25mA; symmetrical biphasic square waves; duration 6 seconds (1.5 second rise and 0.75 second fall); stimulus applied five times per week Bilateral deltoid and quadriceps Application from IMV day 2 to ICU discharge	Hemodynamic variables: HR significantly decreased in G2; no changes in RR before or after intervention in any group
Stefanou et al.^(^^27^^)^	G1: high frequency G2: medium frequency	Total: 36 G1: 18 G2: 18	To investigate the effects of NMES on the mobilization of endothelial progenitor cells among critically ill patients with sepsis	G1: frequency 75Hz, 6 seconds on and 21 seconds off; G2: frequency 45Hz, 5 seconds on and 12 seconds off; biphasic impulse and pulse width 400µs; intensity defined as the maximum tolerated One single 40-minute session Vastus lateralis and peroneus longus	Hemodynamic variables: slight increase of HR and RR; MAP remained the same in both groups. Slight increase in blood lactate in both groups

NMES - neuromuscular electrical stimulation; G - group; IMV - invasive mechanical ventilation; ICU - intensive care unit; COPD - chronic obstructive pulmonary disease; HR - heart rate; RR - respiratory rate; MAP - mean arterial pressure

### Risk of bias

Assessment of the risk of bias based on the method formulated by the Cochrane Collaboration showed that relative to the selection bias aspect of "random sequence generation", two studies exhibited a low risk of bias^(^^23^^,^^25^^)^ and the other two an uncertain risk of bias.^(^^26^^,^^27^^)^ Relative to the selection bias aspect of "allocation concealment", all four articles exhibited an uncertain risk of bias.^(^^23^^,^^25^^-^^27^^)^ In regard to performance bias - "blinding of participants and professionals" - three studies exhibited an uncertain risk of bias^(^^23^^,^^25^^,^^27^^)^ and one study presented a low risk of bias.^(^^26^^)^

For detection bias - "blinding of outcome assessors" - two studies exhibited an uncertain risk of bias^(^^25^^,^^27^^)^ and the other two a low risk of bias.^(^^23^^,^^26^^)^ In regard to attrition bias - "incomplete outcome data" - all four studies^(^^23^^,^^25^^-^^27^^)^ exhibited a low risk of bias. Relative to reporting bias - "selective reporting" - all four studies^(^^23^^,^^25^^-^^27^^)^ exhibited a low risk of bias. Concerning other sources of bias, all four studies^(^^23^^,^^25^^-^^27^^)^ exhibited an uncertain risk of bias.

### Interventions

The studies included in the present review used different comparator groups: one included a control group,^(^^25^^)^ another compared NMES to active mobilization,^(^^26^^)^ a third used the contralateral side of the body as a control,^(^^23^^)^ and the fourth compared two groups subjected to NMES with different frequencies.^(^^27^^)^ In none of the selected studies was the safety of the technique the primary outcome. In the present review, we used the secondary outcomes and corresponding data (Table 2).

Two out of the four included studies assessed NMES safety based on hemodynamic variables. Stefanou et al.^(^^27^^)^ found significant differences in heart rate, respiratory rate and blood lactate, which were not considered clinically relevant. In contrast, Akar et al.^(^^26^^)^ did not find any significant differences between the groups.

The other two studies assessed safety based on reported adverse effects. In the study by Abu-Khaber et al.,^(^^25^^)^ 15% of the participants described a prickling sensation, which was not clinically significant. In the study by Rodriguez et al.,^(^^23^^)^ there was one case of a superficial burn due to the improper configuration of NMES parameters.

## DISCUSSION

The present systematic review, based on RCTs, found that as a means to prevent ICU-acquired muscle weakness and in comparison to a control group, NMES is safe provided it is properly applied by a duly trained professional.

None of the studies included in the present review assessed the safety of the technique of interest as the main outcome. However, they assessed variables able to detect risk in the application of NMES.

The ideal dose for use in NMES training protocols has not yet been established, as several systematic reviews on this subject show that there is wide variation in the intensity, duration, number of repetitions and site of application.^(^^23^^,^^27^^)^ On these grounds, one should consider the hypothesis that patients might be undertreated, this being the cause for the lack of reports of adverse effects.

The population in the study by Rodriguez et al.^(^^23^^)^ exhibited sepsis, which is a common occurrence in the ICU associated with systemic inflammation, which is an inducer of protein catabolism. The authors detected one case of skin burn following a session in which the configurations did not comply with the predefined protocol. Other studies conducted with patients with sepsis did not report any adverse effects among the patients subjected to NMES.^(^^9^^)^

A prickling sensation was the only complication described by 15% of the patients subjected to NMES in the study by Abu-Khaber et al.,^(^^25^^)^ which was included in the present systematic review. According to the authors, this occurrence was no reason to limit the intervention. In turn, Fischer et al.^(^^28^^)^ detected five cases of patients who reported discomfort during the application of NMES, which was no reason to discontinue the intervention; they did not describe any hemodynamic abnormalities. Pain was the reason why one out of 68 patients dropped out of another study.^(^^29^^)^

One of the factors that aggravates the clinical condition of critically ill patients is the intense inflammation they develop. In addition to the state of hypermetabolism triggered by the inflammation, increased protein catabolism and overload of kidney and heart function also occur. Therefore, all situations that enhance the inflammatory response are undesirable. In a study included in the present review, Akar et al.^(^^26^^)^ found a reduction of the inflammatory response for the duration of mechanical ventilation among patients who underwent NMES combined with active exercise and the group that received NMES alone. Interleukin 6 levels decreased in the group that underwent NMES combined with exercise, and interleukin 8 levels decreased in the groups that received NMES alone or in combination with exercise. These findings suggest that NMES is not associated with the risk of an increase of the inflammatory response among critically ill patients. However, the authors did not categorize the study participants as per the severity of their clinical condition or the presence of sepsis.

Akar et al.^(^^26^^)^ further found a significant reduction in heart rate after the intervention. This finding suggests that NMES does not cause cardiac overload. In fact, this finding might denote a clinical improvement and even a cardiovascular adaptation to treatment.^(^^26^^)^ In contrast, Stefanou et al.^(^^27^^)^ found an elevation in heart rate in their sample.

In regard to the deaths that occurred in the included studies, there is no indication they were associated with the use of NMES. In the study by Akar et al.,^(^^26^^)^ mortality was higher (50%) in the group that did not receive NMES, while relative to the two groups that received NMES, patients out of 20 died.

Among 17 RCTs involving the application of NMES to critically ill patients and subjected to full-text analysis in the present review, only four approached patient safety and were included for review. The fact that the other studies did not make mention of adverse effects suggests that this therapeutic strategy has no unhealthy effects for critically ill patients.

Two observational studies and one pilot study assessed the safety of NMES among critically ill patients.^(^^1^^,^^29^^,^^30^^)^

Iwatsu et al.^(^^29^^)^ followed up with 61 patients throughout the postoperative period following heart surgery and analyzed the safety of NMES. Frequencies of 200Hz and 20Hz were alternated, and the intensity of the current was defined in the postoperative period, with patients receiving 10% to 20% of the maximum torque. Safety outcomes were hemodynamic parameters, pacemaker function and arrhythmias. None of these parameters exhibited any abnormalities, which allowed the authors to conclude that NMES does not increase the cardiovascular workload, and thus is safe for the target population.

In an observational study, Segers et al.^(^^1^^)^ assessed blood pressure, heart rate, respiratory rate, oxygen saturation and skin reactions as safety outcomes of the application of NMES to critically ill patients. The participants received the intervention five times per week, with an intensity up to 80mA, pulse duration up to 500ms and frequency of 50Hz. No significant change was detected in the investigated variables; only skin hyperemia occurred in 50% of the patients following removal of the electrodes, which disappeared gradually. There were no reports of pain limiting the intervention.

In a pilot study that compared a group of patients with sepsis under mechanical ventilation who received NMES combined with ergometric cycling versus a control group, Parry et al.^(^^30^^)^ selected safety parameters to determine continuation or discontinuation of NMES: heart rate below 50 or over 140bpm, mean arterial pressure below 65mmHg, need of fraction of inspired oxygen over 80%, need of positive end-expiratory pressure (PEEP) over 15mmHg, respiratory rate over 35 bpm, oxygen saturation below 85% or a 10% fall, and self-reported pain score over 7 on a visual analog scale. The authors did not detect any serious adverse effects, just one case of desaturation 30 minutes after the intervention. Thus, they concluded that NMES was safe among critically ill patients.

One of the main limitations of the present study derives from the methodological diversity among the included studies. The use of the contralateral lower limb as a control in the study by Rodriguez et al.^(^^23^^)^ does not allow the assessment of possible systemic abnormal changes following the application of NMES. In turn, Stefanou et al.^(^^27^^)^ performed one single NMES session, which does not allow assessment of the effects of continued use or the progressive increase of intensity on muscle mass and the cardiovascular system.

## CONCLUSION

Neuromuscular electrical stimulation is a safe technique for application to critically ill patients by duly trained professionals and with proper evidence-based parameters. New randomized clinical trials should be conducted, with the safety of neuromuscular electrical stimulation among critically ill patients as the primary outcome.
